# Opioids and Their Receptors: Present and Emerging Concepts in Opioid Drug Discovery II

**DOI:** 10.3390/molecules27103140

**Published:** 2022-05-13

**Authors:** Richard M. Van Rijn, Mariana Spetea

**Affiliations:** 1 Department of Medicinal Chemistry and Molecular Pharmacology, Purdue Institute for Drug Discovery, and Purdue Institute for Integrative Neuroscience, Purdue University, West Lafayette, IN 47907, USA; 2 Septerna Inc., San Francisco, CA 94080, USA; 3 Department of Pharmaceutical Chemistry, Institute of Pharmacy and Center for Molecular Biosciences Innsbruck (CMBI), University of Innsbruck, 6020 Innsbruck, Austria

A few neurotransmitter systems have fascinated the research community, as much as the opioid system (i.e., opioid ligands and their receptors). Over the years, scientific studies of the endogenous opioid system have uncovered a complex and subtle system that exhibits impressive diversity, based on its critical role in modulating a large number of sensory, motivational, emotional, and cognitive functions. Additionally, its important therapeutic value for the treatment of many human disorders, including pain, affective and addictive disorders, and gastrointestinal motility disorders, has been of persistent interest.

The Special Issue, “Opioids and Their Receptors: Present and Emerging Concepts in Opioid Drug Discovery II”, which follows a similar topical Special Issue published in 2020 [1], includes eleven research articles and three reviews. This Special Issue offers up-to-date and new perspectives about opioid drug discovery.

Three research articles cover the discovery of novel δ-opioid receptor (δOR) ligands with distinct pharmacological profiles [2,3,4,5]. Meqbil et al. identified a novel δOR agonist with a unique scaffold lacking basic nitrogen from a high-throughput screen [4]. Molecular dynamics simulations of the molecule in the presence or absence of a docked Leu^5^-enkephalin peptide suggests that this molecule interacts with δOR in a bitopic manner. Specifically, the molecule partly occupies the orthosteric pocket in which the enkephalin peptide resides, but it also fits in a generally idle subpocket of the binding pocket. Cellular assays indicate that the molecule has a 10-fold preference for binding to the δOR over µ- and κ-opioid receptors (µOR and κOR, respectively), and it competes with orthosteric ligands. However, modeling and competitive functional assays suggest that the molecule may possess some negative modulatory capabilities. The study by Karasawa et al. confirmed previous work by Cassell et al. showing rubiscolin-5 (Tyr-Pro-Leu-Asp-Leu) and rubiscolin-6 (Tyr-Pro-Leu-Asp-Leu-Phe) to selectively bind and activate δORs without recruiting β-arrestin 2 [2,6]. The authors noted significant changes in the efficacy of rubiscolin peptides to inhibit intracellular cAMP in cells co-expressing δOR and µOR, potentially indicating an affinity for putative δOR-µOR heteromers; however, this type of assay comes with multiple limitations in terms of controlling receptor expression and dissecting the cAMP signal that originates from the monomers, this could be better resolved in a model system that eliminates monomer signaling [7]. Tanguturi et al. reported on a couple of novel δOR inverse agonists [3,5]. This work was inspired by a prior study by Higashi et al. [8] and identified SRI-9342 as an irreversible antagonist and SRI-45128 as an inverse agonist. The high affinity and selectivity for δOR over µOR and κOR make these valuable tools, which could, for example, be used to investigate the utility of this class of δOR modulators in treating Alzheimers’ disease. Similarly to the study by Karasawa et al., one exciting strength of the study by Tanguturi et al. is that it confirms findings by a different research team, providing much greater validity to the unique pharmacology, be it a G protein-biased peptide or an inverse agonist.

Wtorek et al. presented a continuation of their work on pentapeptide Tyr-c[D-Lys-Phe-Phe-Asp]NH_2_ (RP-170), a stabilized bifunctional µOR and κOR agonist with central and peripheral antinociceptive properties [9]. In the current study [10], D-Lys was replaced with either an (*R*)-β^3^-Lys (RP-171) or a (*S*)-β^3^-Lys (RP-172). Both RP-171 and RP-172 lost affinity and potency relative to the parent compound, with RP-172 precipitously so. However, RP-171 gained µOR selectivity in both affinity (14-fold from 3-fold) and potency (7-fold from 2-fold) relative to RP-170. Molecular dynamics simulations suggested that RP-172 was less able to form or maintain hydrogen bonds and a salt bridge with Asp147.

Yucel et al. designed and synthesized novel molecules with thiazole and piperazine moieties [11], based on the rationale that many analgesic drugs, such as for example amoxapine and meloxicam, carry these structural motifs. Multiple synthesized molecules produced antinociception in mouse models of acute nociceptive (tail-clip and hot-plate tests) and visceral pain (acetic acid-induced writhing test) following oral administration. The authors found the effects to be naloxone reversible, which is suggestive of an opioid receptor mechanism. Molecular docking studies predict that the molecules can product meaningful interactions within the µOR and δOR binding pocket, whereas docking scores for the molecules within the κOR structure did not correlate with behavioral efficacy, i.e., inactive derivatives docked equally as active derivatives.

A study by Fritzwanker et al. examines µOR phosphorylation and dephosphorylation by SR-17018 compared to the canonical agonist DAMGO and the partial agonist buprenorphine [12]. The authors observed that SR-17018 has a delayed onset of µOR phosphorylation, but it otherwise matches the full agonist profile of phosphorylating µOR at multiple sites. Unlike the full agonist DAMGO, SR-17018-induced µOR phosphorylation persists and is resistant to washout suggestive of a slow off-rate that is, nevertheless, naloxone reversible. SR-17018 has been demonstrated to have a large therapeutic window between antinociception and respiratory depression [13], although there is a debate whether this profile is caused by the G protein bias [14,15]. The findings in this study suggest that SR-17018 clearly has a distinct binding mode that may begin to explain the opioid’s pharmacology.

Other studies in this issue also explored the behavioral pharmacology of opioids in rodent models but outside of their antinociceptive properties. A study by Paul et al. investigated the development of tolerance to the locomotor effects of morphine after twice daily injection (b.i.d.) for a 10-day period [16]. The authors found significant hyperactivity on day 10 relative to day 1. The authors also reveal that tolerance induced by b.i.d. 10 mg/kg morphine treatment was reversed by switching to a 20 mg/kg q.d. dosing regimen. As the authors also tracked the establishment of antinociceptive tolerance, they were able to link antinociceptive tolerance switch to morphine-induced hyper-excitatory activity.

Targeting the κOR receptor is currently regarded as a viable strategy for developing pharmacotherapies for human disorders where the endogenous kappa opioid system (κOR/DYN) plays a central role, including pain, itch, neurological, and addictive disorders [17,18,19]. κOR agonists are under consideration for their antipruritic activity and one such agonist, nalfurafine, is approved in Japan for the treatment of resistant pruritus in hemodialysis patients [20], whereas in the United States, the peptide difelikefalin was approved to treat moderate-to-severe pruritis in the same patient population [21]. Nalbuphine is a third κOR agonist that is being clinically investigated as potential anti-pruritic agent [22]. In a report by Inan et al., in this Special Issue, a more detailed investigation on the antipruritic effects of nalbuphine is presented [23]. The authors tested nalbuphine at multiple doses (0.3–10 mg/kg) in three different acute itch mouse models of TAT-HIV-1 protein, deoxycholic acid, and chloroquine-induced scratching. Nalbuphine dose-dependently inhibited scratching in all three models. The authors also showed that nalbuphine is inactive in the chloroquine model when performed in κOR-knockout mice.

In this issue, Nosova et al. provided a review of epigenetic and transcriptional control of the prodynorphin (*PDYN*) gene in the human brain [24]. The review provides a detailed analysis of different mRNAs produced from the *PDYN* gene as well splice variants and single nucleotide polymorphisms and the potential role of non-coding RNAs. Some of the protein products may serve as nuclear proteins that can impact gene transcription and epigenetic processes. The authors discuss possible transcription factors that can modulate the expression of the *PDYN* gene and the link of SNPs to differential regulation of prodynorphin expression in different neurological disorders. The authors review methylation patterns and discuss differential expressions of *PDYN* between neurons and glia. This review is a highly valuable resource and reference for researchers studying the pDYN/κOR system.

The availability of high-resolution crystal structures of all opioid receptors in active and inactive conformations offer a unique prospect for drug discovery, and has been a significant development for opioid research [25]. Multiple articles in this issue [4,10,11,26] utilized the power of computational techniques (molecular modeling and molecular dynamics simulations) to explore binding mechanisms of peptides and synthetic molecules under investigation using the crystal structures of the opioid receptors. The study by Yucel et al. provides an example of a phenotypic screen where molecular docking aided the investigation into the mechanism of action of the molecules bearing thiazole and piperazine moieties in producing opioid receptor-mediated antinociception [11].

Spetea et al. reported earlier on HS-731 as a full agonist at µOR and δOR, and a partial agonist at κOR [27]. Performing a structure-based molecular modeling study including molecular dynamics simulations and generation of dynamic 3D pharmacophore models (dynophores), Puls et al. provided important insights into dynamic interaction patterns of HS-731 with all opioid receptors [26]. The in silico study nicely rationalizes the experimental results on different binding and activity of HS-731 to each opioid receptor subtype. Two residues are highlighted for HS-731 recognition at µOR, δOR, and κOR, particularly the conserved residue 5.39 (K) and the non-conserved residue 6.58 (µOR: K, δOR: W and κOR: E). At µOR, HS-731 takes part in more frequent and stronger charge interactions than in δOR and κOR, in correlation with the highest affinity of HS-731 at µOR. A salt bridge between transmembrane helices 5 and 6 via K227^5.39^ and E297^6.58^ was postulated to be responsible for the κOR partial agonism of HS-731. Additionally, the lack of binding at the NOP receptor experimentally determined is rationalized by the morphinan phenol Y130^3.33^.

Since the discovery of the NOP receptor as the fourth member of the opioid receptor family, its role in different physiological and pathophysiological processes, especially pain, and the development of potential pain therapeutics was increasingly explored [28] This issue contains a review by El Daibani and Che, highlighting the analgesic utility of the nociception/orphanin FQ receptor (NOP) system [29]. The authors provide a detailed overview of almost two dozen NOP ligands and underscored the need for more high-resolution structures to be resolved beyond the current three crystal structures of the NOP receptor. The authors also touch upon some of the complex behavioral pharmacology observed for NOP agonists depending on whether the animal is administered to rodents or non-human primates at spinal or supraspinal sites. The authors conclude that more studies into the NOP system are necessary, but that the therapeutic promise of NOP agonists as analgesics with reduced risk for respiratory depression persists.

Three articles in this Special Issue explore dimerization and intracellular interactions and positive or negative cooperativity between the µOR and angiotensin (AT2) receptors [30], serotonin (5HT_1A_) receptor [31], and free fatty acid (FFA) receptors [32]. Kiraly et al. reviewed positive cooperativity between µOR analgesics and angiotensin receptor inhibition [30]. The premise of the review is based on studies, for example, that found angiotensin-converting enzyme inhibition enhancing morphine antinociception and reducing opioid antinociceptive tolerance [33] and that the activation of angiotensin AT2 receptor decreases morphine antinociception [34]. Only a handful studies have investigated the interplay between µOR and the angiotensin system, and some of the results have been contradictory. Thus, further studies will be welcome to provide better insight into possible interactions and whether they can be exploited therapeutically.

Binienda et al. investigated but did not identify the presence of a synergistic interaction between the opioid receptor agonists and modulators of FFA receptors [32]. Specifically, the authors tested the µOR agonist DAMGO with FFAR2 antagonist GLPG-09734, FFAR4 agonist GSK 137647, and FFAR4 antagonist AH-7614 in a mouse model of colitis. The FFAR4 antagonist was also tested in the presence of the δOR agonist DPDPE but also without a strong effect. Finally, Radoi et al. utilized fluorescence cross-correlation spectroscopy to examine whether the opioids morphine, codeine, oxycodone, and fentanyl promoted heterodimerization between the serotonin 5HT_1A_ receptor and µOR [31]. The authors further assessed the ability of the four opioids to stimulate ERK1/2 and p38 phosphorylation in cells co-expressing µOR and 5HT_1A_ receptors. While the authors noted differences in phosphorylation strength MAPK subtype, the experimental design limited the conclusions that could be drawn from those findings. Since 5HT_1A_ receptors may have roles in nociception, the further examination of the 5HT_1A_R-µOR interaction may provide novel strategies to promote the effectiveness of opioid analgesics.

The final collection of articles in this Special issue covers a broad area of opioid research that encompass all four opioid receptors; in silico, in vitro, and in vivo approaches; and small molecules and peptide ligand design. Therefore, we are optimistic that there will be relevant and useful articles amongst the collection to suit any scientist or member of the public regardless of their specific research focus or interests.

We would like to thank all authors for their contributions to this second edition of the Special Issue covering current and emerging concepts in opioid drug discovery. We also thank all reviewers for their effort in evaluating the manuscripts. Last but not least, we would like to appreciate the editorial office of the *Molecules* journal for their support in preparing this Special Issue.

## Data Availability

Not applicable.
